# Supplementary material for: Glioma stem cells invasive phenotype at optimal stiffness is driven by MGAT5 dependent mechanosensing
Source: J Exp Clin Cancer Res. 2021 Apr 24;40:139. doi: 10.1186/s13046-021-01925-7 (PMC8067292; doi:10.1186/s13046-021-01925-7)
Supplement: Supplementary file 1 — Additional file 1: Figure S1. Atomic Force Microscopy force-volume maps. (A, B, C, D) 3D reconstructions of height of the contact point of control, 0.0015% w/w of MWCNT, 0.00625% w/w and 0.05% w/w samples, respectively. Histograms of the associated Young’s modulus E fitted with a Gaussian distribution. Stiffness values given in the text correspond to the mean ± SD of 5 independent elasticity maps. Statistical significance was determined using one-way ANOVA with post hoc Tukey’s Honest Significant Difference test for multiple comparisons. P values of less than 0.05 were considered significant. (D) 3D-reconstruction of the contact point’s height. Figure S2. Western Blot quantification of WT GSC proteins expression. Each western blot quantification was performed three to seven times and normalized with respect to GAPDH expressions and sum. Bands were quantified using the Bio-Rad Chemidoc and the image lab software. Data are presented as mean +/− SEM and statistical significance was determined using one-way ANOVA (* p < 0,05; ** p < 0,01; *** p < 0,005; **** p < 0,001). Figure S3. Examples of MS analysis and quantification of fucosylated glycans. Glycans were isolated from wild type (WT) or MGAT5 KO (1.2) GSCs cultivated either as neurospheres (NS) in proliferation medium or as adherent cells (ADH) in 2D in differentiation medium. The minor structures with identical masses, group 2 and 1 are likely to be isomers, for example two linear N-acetyllactosamine units on a bi- rather than tri – antennary N-glycan. (A, B) MS analysis and quantification tri-antenate glycancs with a fucosylated core of. (B, C, D) MS analysis and quantification of tri-antenate glycans di-fucosylated on the branches. (E, F) MS analysis and quantification tetra-antenate glycans mono fucosylated on the branches. Data are presented as mean +/− SEM. Figure S4. Western Blot quantification of MGAT5 KO GSC proteins expression. Each western blot quantification was performed three to four times and normaliz [file 13046_2021_1925_MOESM1_ESM.docx]

**Supplementary figure 1**

**
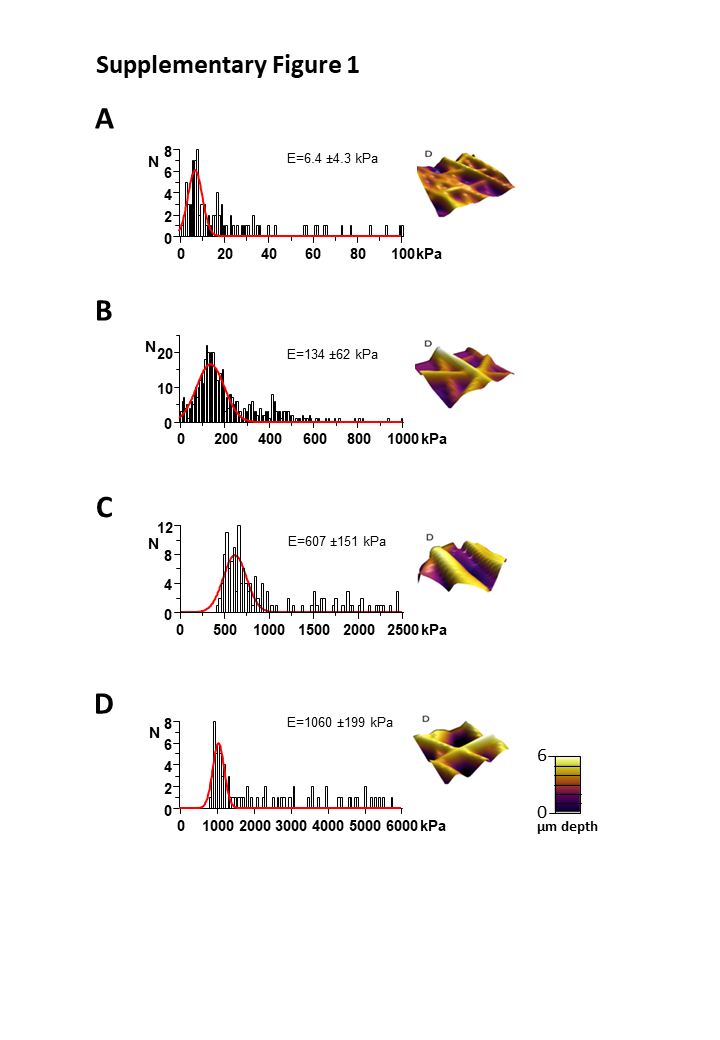
**

**Supplementary figure 1: Atomic Force Microscopy force-volume maps.**

(A, B, C, D) 3D reconstructions of height of the contact point of control, 0.0015% w/w of MWCNT, 0.00625% w/w and 0.05% w/w samples, respectively. Histograms of the associated Young’s modulus E fitted with a Gaussian distribution. Stiffness values given in the text correspond to the mean ± SD of 5 independent elasticity maps. Statistical significance was determined using one-way ANOVA with post hoc Tukey’s Honest Significant Difference test for multiple comparisons. P values of less than 0.05 were considered significant. (D) 3D-reconstruction of the contact point’s height.

**Supplementary figure 2**

**
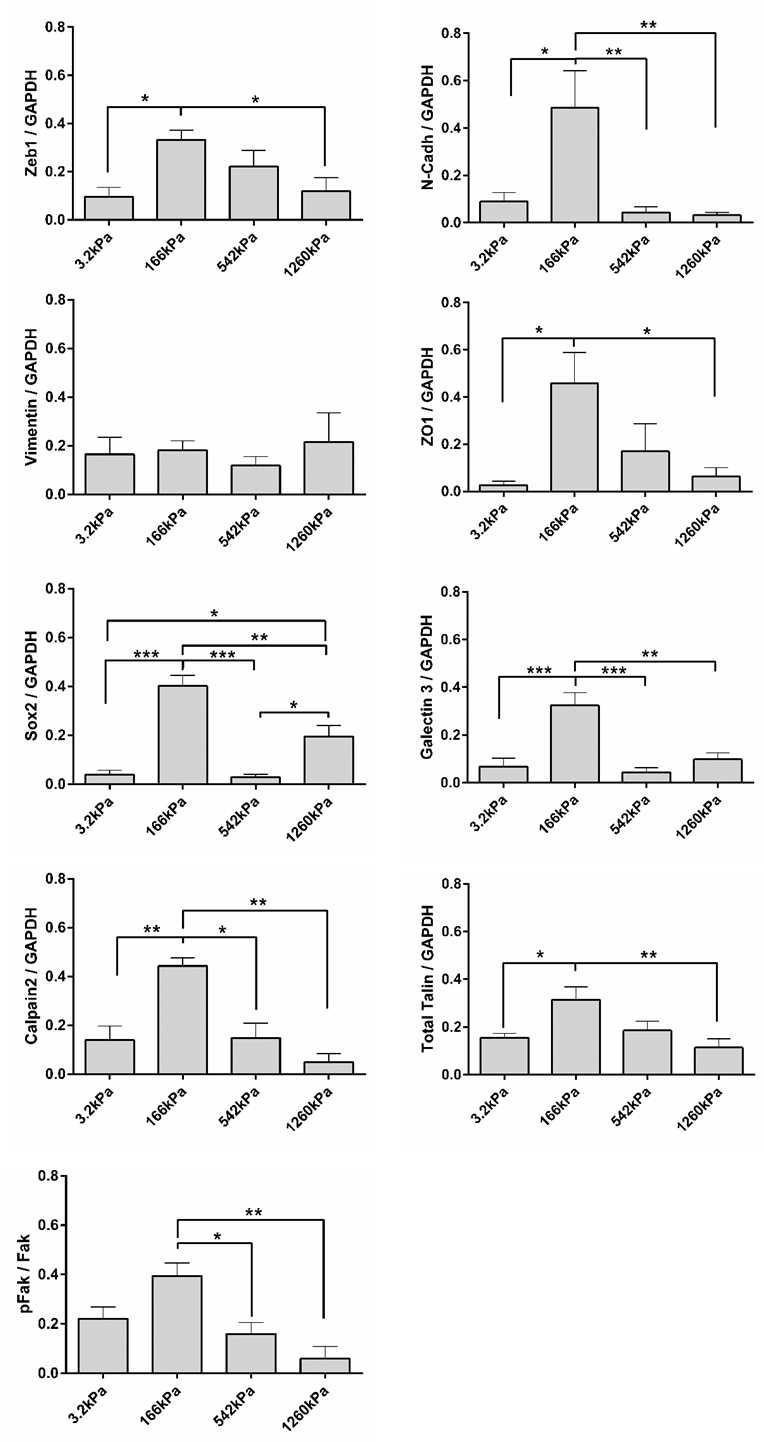
**

**Supplementary figure 2: Western Blot quantification of WT GSC proteins expression.** Each western blot quantification was performed three to seven times and normalized with respect to GAPDH expressions and sum. Bands were quantified using the Bio-Rad Chemidoc and the image lab software . Data are presented as mean +/- SEM and statistical significance was determined using one-way ANOVA (* p<0,05; ** p<0,01; *** p<0,005; **** p<0,001).

**Supplementary figure 3**

**
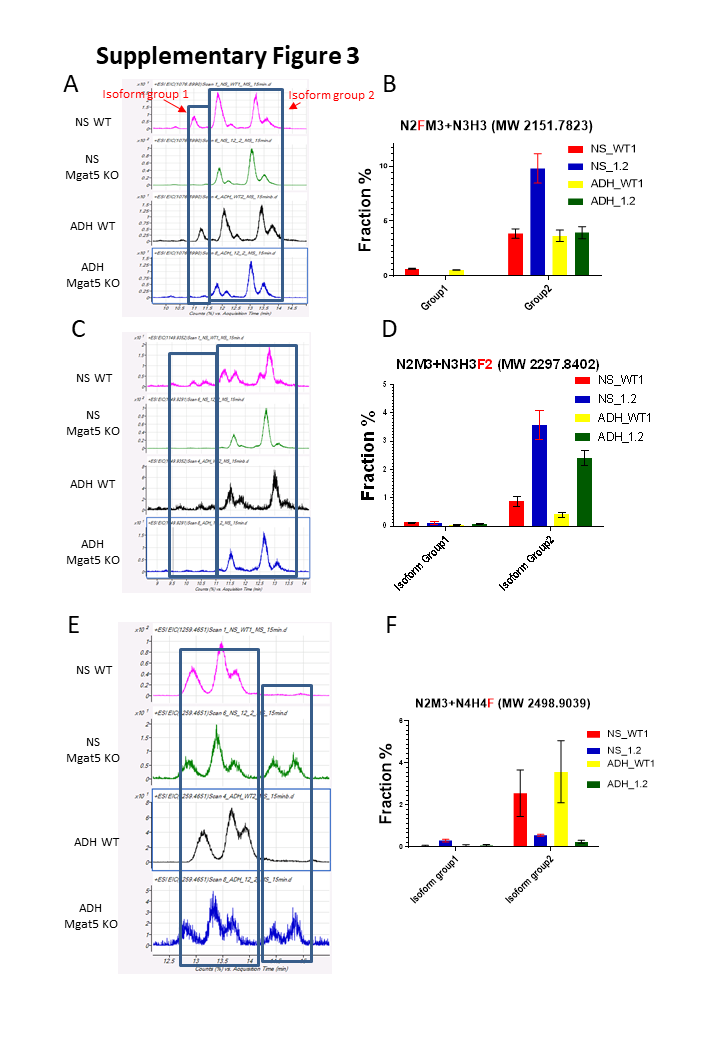
**

**Supplementary figure 3: Examples of MS analysis and quantification of fucosylated glycans.** Glycans were isolated from wild type (WT) or MGAT5 KO (1.2) GSCs cultivated either as neurospheres (NS) in proliferation medium or as adherent cells (ADH) in 2D in differentiation medium. The minor structures with identical masses, group 2 and 1 are likely to be isomers, for example two linear N-acetyllactosamine units on a bi- rather than tri – antennary N-glycan. (A, B) MS analysis and quantification tri-antenate glycancs with a fucosylated core of . (B, C, D) MS analysis and quantification of tri-antenate glycans di-fucosylated on the branches. (E, F) MS analysis and quantification tetra-antenate glycans mono fucosylated on the branches. Data are presented as mean +/- SEM

**Supplementary figure 4**

**
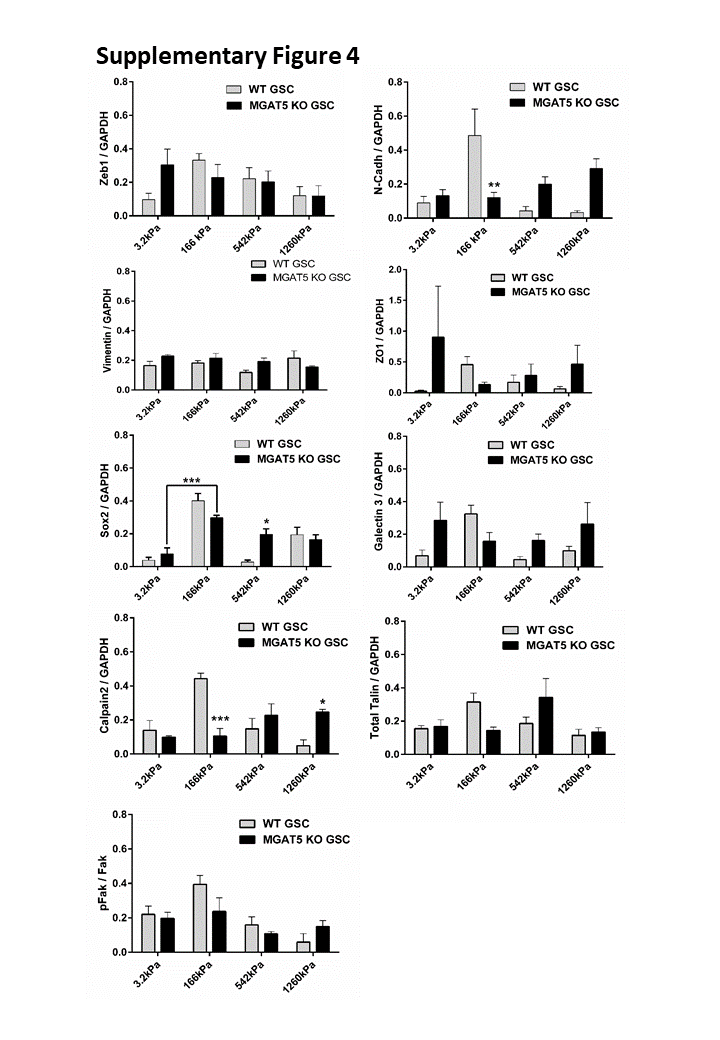
**

**Supplementary figure 4: Western Blot quantification of MGAT5 KO GSC proteins expression.** Each western blot quantification was performed three to four times and normalized with respect to GAPDH expressions and sum. Bands were quantified using the Bio-Rad Chemidoc and the image lab software . Data are presented as mean +/- SEM and statistical significance was determined using one-way ANOVA (* p<0,05; ** p<0,01; *** p<0,005; **** p<0,001).

**Supplementary Figure 5**

**
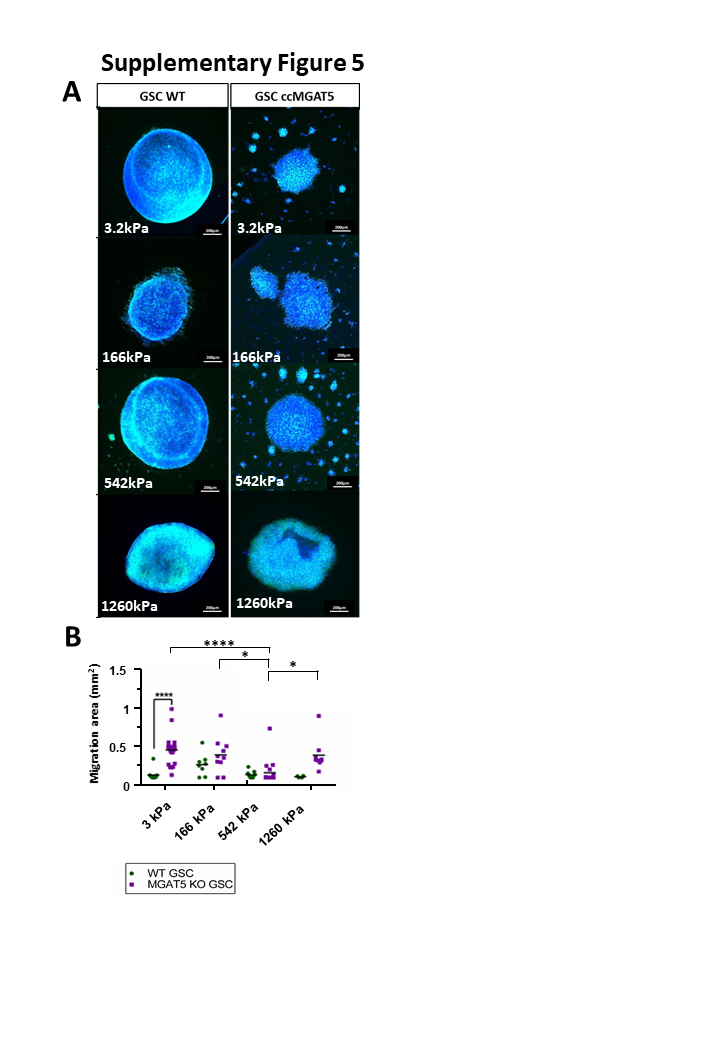
**

**Supplementary figure 5: MGAT5 KO GSC migration in proliferation medium compared to WT GSCs.** (A) Pictures of WT and MGAT5 KO WT GSCs migration behaviour in proliferation medium of WT GSCs at every stiffnesses. Nucleus stain with Hoechst 33342. (B) Quantification 5 days after NS plating of migration area (μm²) in the proliferation condition of WT GSCs and MGAT5 KO GSCs. Data are presented as mean +/- SEM and statistical significance was determined using one-way ANOVA (* p<0,05; ** p<0,01; *** p<0,005; **** p<0,001).
